# Tyr724 phosphorylation of ELMO1 by Src is involved in cell spreading and migration *via* Rac1 activation

**DOI:** 10.1186/s12964-015-0113-y

**Published:** 2015-07-25

**Authors:** Yoshinori Makino, Masumi Tsuda, Yusuke Ohba, Hiroshi Nishihara, Hirofumi Sawa, Kazuo Nagashima, Shinya Tanaka

**Affiliations:** Department of Cancer Pathology, Hokkaido University Graduate School of Medicine, N15, W7, Kita-ku, Sapporo, 060-8638 Japan; Laboratory of Pathology and Development, Institute of Molecular and Cellular Biosciences, The University of Tokyo, Tokyo, 113-0032 Japan; Department of Cell Physiology, Hokkaido University Graduate School of Medicine, N15, W7, Kita-ku, Sapporo, 060-8638 Japan; Department of Translational Pathology, Hokkaido University Graduate School of Medicine, N15, W7, Kita-ku, Sapporo, 060-8638 Japan; Hokkaido University Research Center for Zoonosis Control, Sapporo, 001-0020 Japan; Sapporo Higashi Tokushukai Hospital, Sapporo, 065-0033 Japan

**Keywords:** ELMO1, Src, Dock180, Tyrosine phosphorylation, Cell spreading, Migration

## Abstract

**Background:**

The complex of Dock180/ELMO1 that functions as a bipartite guanine nucleotide exchange factor for Rac is essential for diverse physiological and pathological processes of cells such as cell migration, phagocytosis, and invasion of cancer cells. Among the Src-family tyrosine kinases (SFKs), it has been reported that Hck directly phosphorylates ELMO1, regulating phagocytosis by promoting activation of Rac1; however, the involvement of other SFKs in ELMO1 phosphorylation has remained unknown. Here, we identified novel tyrosine (Y) residues of ELMO1 phosphorylated by SFKs, and examined the effects on Rac1 activity, cell adhesion, spreading, and cell motility on extracellular matrix (ECM).

**Results:**

In this study, we unveiled that Src and Fyn can induce tyrosine phosphorylation of ELMO1 in *in vivo* and *in vitro* phosphorylation assays. Mutational analyses identified both Y720 and Y724 residues of ELMO1 as Src-mediated phosphorylation sites, preferentially on Y724. Single substitution of Y724 to Phe abrogated Rac1 activation triggered by Src. To elucidate the biological function of pY724, we established NIH3T3 cells stably expressing wild-type ELMO1 or its Y724F mutant together with Dock180. Among them, Y724-deficient cells exhibited a depletion of Rac1 activity with diminished phosphorylation of ELMO1 even upon the ECM-stimulation. It is noteworthy that NIH3T3 cells with ELMO1 Y724F were strikingly defective to promote cell spreading on fibronectin-coated dish, concomitantly exhibiting immature assemblies of actin stress fibers and focal adhesions. Eventually, ELMO1 Y724F significantly impaired cell migration.

**Conclusion:**

These results define that Src-mediated Y724 phosphorylation in ELMO1 plays a critical role for cell spreading *via* activation of Rac1, leading to promotion of cell migration. As the overexpression and/or hyperactivation of Src have been shown in a wide variety of human cancers, Src-mediated phosphorylation of Y724 in ELMO1 may regulate cancer cell adhesion to the ECM, invasion into surrounding tissues, and subsequent distant metastasis.

**Electronic supplementary material:**

The online version of this article (doi:10.1186/s12964-015-0113-y) contains supplementary material, which is available to authorized users.

## Background

Cell migration is essential for a wide variety of physiological and pathological processes, including embryonic development, wound repair, inflammatory responses, and tumor metastases. Signalling adaptor protein Crk that is bound to Dock180 (downstream of Crk with 180 kDa) has been shown to be one of the essential molecules to regulate focal adhesion. Tyrosine kinases including Fak or components of focal adhesion such as p130^Cas^ are conventional upstream regulators of Dock180 through the binding to Crk. Currently, the trimolecular complex known as Crk/Dock180/ELMO1 (regulator of engulfment and motility 1) complex plays a pivotal role in cell migration [1, 2]. ELMO1 was originally identified as the Dock180 binding protein regulating engulfment and cell migration in nematodes, and currently it is known that Dock180/ELMO1 functions as a bipartite guanine nucleotide exchange factor (GEF) for the small GTPase Rac1 and regulates cytoskeletal remodeling [3].

ELMO1 possesses a pleckstrin homology (PH) domain and proline-rich motif residing in its C-terminus [4, 5], but has no catalytic domain. Through its proline-rich motif, ELMO1 binds to the SH3 domain of Dock180 and enhances GEF activity of Dock180 by relieving the inhibitory loop of Dock180 *per se* [6]. It has been recently reported that the atypical PH domain of ELMO1 directly interacts with Dock180 in a Rac-independent and constitutive manner [7]. In addition, small GTPase RhoG directly binds to Armadillo (ARM) repeats of ELMO1 at the N-terminus, and the ternary complex comprised of RhoG, ELMO1, and Dock180 may activate Rac1 at the plasma membrane, resulting in integrin-mediated cell spreading, phagocytosis, and nerve growth factor (NGF)-induced neurite outgrowth [8, 9].

ELMO1/Dock180 complex is implicated in pathogenesis of various diseases such as diabetic nephropathy, HIV infection, and tumor development [10, 11]. Especially, enhanced expression of ELMO1/Dock180 complex together with Crk is evidently linked to the invasiveness of brain tumors [12] and ovarian cancers [13]. Protein levels of Dock180 are possibly controlled by ELMO1, in which ELMO1 inhibits ubiquitylation of Dock180 and prevents proteasome-dependent degradation of Dock180 through the direct interaction [14].

Although ELMO1 is essential for the activation of Dock180, the mechanism regulating the activity of ELMO1 has remained obscure. Intriguingly, it has been reported that ELMO1 directly binds to the SH3 domain of hematopoietic cell kinase (Hck), a member of the Src family of protein tyrosine kinases (SFKs) *via* its proline-rich motif, and the subsequent phosphorylation on Y511 of ELMO1 by Hck plays an important role in cell migration and phagocytosis [15, 16]. Recently, the receptor tyrosine kinase Axl also phosphorylates ELMO, and promotes Rac activation and cell invasion [17].

SFKs are non-receptor tyrosine kinases involved in various signalling pathways including cell proliferation, migration, adhesion, and angiogenesis [18, 19]. Overexpression and/or activation of Src have been frequently detected in a variety of tumors arising from the lung, breast, colon, prostate, and pancreas [18, 20], and hyperactivity of Src is correlated with tumor progression, metastasis, and poor prognosis [21]. These evidences indicate the significance of Src-mediated signalling pathways in the development and progression of human cancers.

In this study, we demonstrated that Src contributed to tyrosine phosphorylation of ELMO1 at Y720 and Y724 residues, which was crucial for activation of Rac1, followed by the promoting cell adhesion, spreading, and migration.

## Results

### Tyrosine phosphorylation of ELMO1 by SFKs

**Fig. 1:**
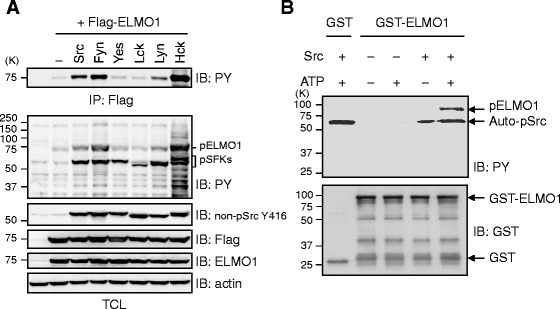
Phosphorylation of ELMO1 by Src. **a** *In vivo* phosphorylation of ELMO1 by SFKs. HEK293T cells were transfected with pCXN2-Flag-ELMO1 in combination with plasmids expressing SFKs: Src, Fyn, Yes, Lck, Lyn, and Hck. Lysates from these cells were subjected to immunoprecipitation with anti-Flag antibody (Ab) for ELMO1, followed by immunoblotting with Ab to phosphotyrosine (PY). Anti-non-phospho-Src Y416 antibody recognizes conserved region of other SFKs such as Lyn, Fyn, Lck, Yes, and Hck. IP: immunoprecipitation, TCL: Total Cell Lysate, pELMO1: tyrosine phosphorylated ELMO1, pSFKs: phosphorylated SFKs. **b** *In vitro* phosphorylation of ELMO1 by Src. Recombinant proteins of c-Src and GST-ELMO1 were mixed with or without ATP for kinase reaction. Reaction samples were analyzed by immunoblotting with Abs against PY (upper) and GST (lower). Auto-pSrc: autophosphorylated Src

To explore a potential of SFKs on ELMO1 phosphorylation, each of SFK such as Src, Yes, Fyn, Lyn, Lck, and Hck was co-expressed with ELMO1 in 293 T cells. Anti-non-phospho-Src Y416 antibody recognizes conserved region of SFKs, which certified that equivalent amounts of SFKs such as Src, Fyn, Yes, Lck, Lyn, and Hck were overexpressed in 293 T cells (Fig. 1a). Under this condition, ELMO1 was remarkably phosphorylated in the presence of Src and Fyn, in addition to Hck (Fig. 1a), compared to the other kinases. Magnitude of tyrosine phosphorylation of ELMO1 is not completely correlated to the tyrosine phosphorylation levels of total cell lysates (Fig. 1a, lower panel). As Src has been reported to be overexpressed in several human cancers, we focused on Src and examined whether Src directly phosphorylates ELMO1 by *in vitro* kinase assay using GST-ELMO1 as a substrate. ELMO1 could be tyrosine-phosphorylated by Src without an association with Dock180 (Fig. 1b).

### Tyrosine 724 of ELMO1 is a phosphorylation site by Src

**Fig. 2:**
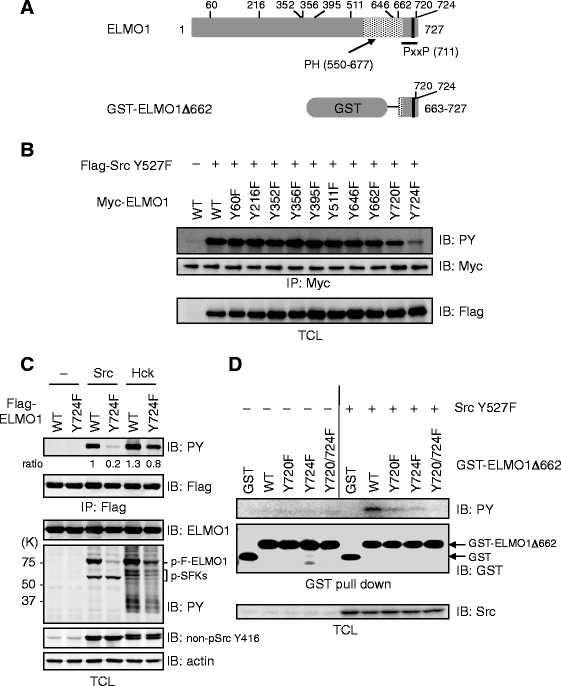
Tyr724 residue of ELMO1 is a phosphorylation site by Src. **a** Schematic representations show ELMO1 protein with the tyrosine residues mutated (from tyrosine to phenylalanine residue, Y60F, Y216F, Y352F, Y356F, Y395F, Y511F, Y646F, Y662F, Y720F, Y724F, upper), and its deletion mutant (663–727 amino acids) fused with GST (lower). PH: Pleckstrin Homology domain, PxxP: proline-rich motif. **b** *In vivo* phosphorylation of ELMO1 mutants by Src. HEK293T cells were transfected with either pCMV-Myc-ELMO1 or its defective mutants on tyrosine, together with pCXN2-Flag-Src Y527F. Lysates from these cells were subjected to immunoprecipitation with anti-Myc Ab for ELMO1, followed by immunoblotting with Abs to PY (upper) and Myc (middle). Expression of extrinsic Src was also examined using anti-Flag Ab (lower). **c** *In vivo* phosphorylation of ELMO1 Y724F mutant by Src and Hck. Lysates from HEK293T cells expressing Flag-tagged ELMO1 and its Y724F mutant in the presence or absence of Src and Hck were subjected to immunoprecipitation and immunoblot analyses. **d** *In vivo* phosphorylation of ELMO1Δ662 by Src. HEK293T cells were transfected with pEBG-ELMO1Δ662 and its mutant forms (Y720F, Y724F, Y720/724 F), together with pMik-Src Y527F. Lysates from the cells were subjected to the pull-down assay with glutathione-sepharose beads, followed by immunoblotting with Abs to PY (top) and GST (middle). Expression levels of Src (intrinsic Src and its extrinsic Y527F) were examined using anti-Src Ab (bottom). GST-ELMO1Δ662: ELMO1Δ662 fused with GST

To identify the Src-mediated phosphorylation site of ELMO1, we constructed a series of mutants of ELMO1. Among 19 tyrosine residues in ELMO1, ten of them (Y60, 216, 352, 356, 395, 511, 646, 662, 720, and 724) satisfy criteria for potential Src-, but not SFKs-, dependent phosphorylation sites according to the prediction service of phosphorylation (NetPhos service, CBS Prediction Servers, Technical Univ. of Denmark, http://www.cbs.dtu.dk/services/NetPhos/, Additional file 1: Table S1). Thus, these tyrosine residues of ELMO1 were individually substituted by phenylalanine (Fig. 2a, upper) and coexpressed with a constitutively active form of Src (Src Y527F) in 293 T cells. Tyrosine phosphorylation levels of ELMO1 Y724F mutant was markedly reduced compared to that of wild-type ELMO1 (Fig. 2b). The phosphorylation level of the Y720F mutant was also attenuated, albeit to a lesser extent, whereas phenylalanine substitution for other tyrosine residues of ELMO1 showed no reduction of phosphorylation (Fig. 2b). It should be noted that Src-dependent tyrosine phosphorylation of ELMO1 Y511F was not decreased (Fig. 2b), although this Y511 residue has been reported to be phosphorylated by Hck [15]. Src-mediated phosphorylation of ELMO1 was remarkably decreased by the Y724F mutation (Fig. 2c). As Hck-dependent phosphorylation of ELMO1 was also partially decreased by Y724F mutation (Fig. 2c), which suggests that Hck can phosphorylate Y724, in addition to previously identified pY site as Y511.

To elucidate the mechanism underlying phosphorylation of ELMO1 by Src, we generated a deletion mutant of ELMO1 lacking N-terminal 662 amino acids (GST-ELMO1Δ662; Fig. 2a, lower panel). ELMO1Δ662 was phosphorylated by activated SrcY527F (Fig. 2d). Thereafter, two tyrosine residues Y720 and Y724 in GST-ELMO1Δ662 were substituted by phenylalanine individually or simultaneously (GST-ELMO1Δ662-Y720F, Δ662-Y724F, and Δ662-Y720/724 F). In single substitutional mutant such as Y720F or Y724F, the phosphorylation levels remarkably decreased but still remained, whereas their double mutant as Y720F/Y724F completely abrogated the phosphorylation, suggesting the both Tyr residues can be phosphorylated by Src (Fig. 2d).

### Requirement for Y724 phosphorylation of ELMO1 in Src-dependent Rac1 activation

**Fig. 3:**
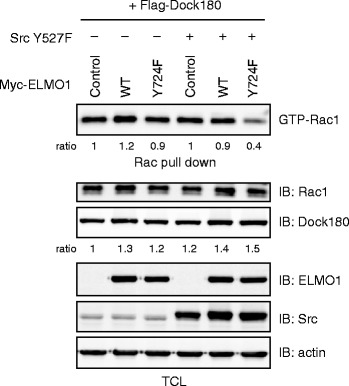
Involvement of ELMO1 pY724 on Rac1 activation triggered by Src. Plasmids for expressing Flag-Dock180, Src Y527F, Myc-ELMO1 and its Y724F mutant were introduced in HEK293T cells as indicated. The lysates were subjected to pull-down assay for Rac1 activity. Expression levels of total Rac1, Dock180, ELMO1, and Src were also examined by immunoblotting. Actin was a loading control. This experiment was repeated for 4 times

We next examined the contribution of ELMO1 phosphorylation to Rac1 activation. Under an intrinsic expression of Src in 293 T cells, co-forced expressions of Dock180 and wild-type ELMO1 enhanced Rac1 activity (Fig. 3, lane 1 vs 2) as reported previously [3]. This activation was not markedly affected by Y724F substitution of ELMO1 (Fig. 3, lane 3), nor by all of the other substitutions (Additional file 2: Figure S1). Meanwhile, upon an introduction of active SrcY527F (Fig. 3, lanes 4–6), forced expression of wild-type ELMO1 could not enhance Rac1 activity (Fig. 3, lane 4 vs 5), due to presumably maximum activation of endogenous Rac1 upon Src activation. In this context, ELMO1-Y724F significantly suppressed Rac1 activity (Fig. 3, lane 6), suggesting the pivotal role of pY724 of ELMO1 in Src-triggered activation of Rac1. Of note, the amount of Dock180 protein was up-regulated in the presence of exogenous ELMO1 (Fig. 3), which may be due to inhibition of ubiquitination of Dock180 through the interaction with ELMO1, as we have reported previously [14].

### Establishment of NIH3T3 cell lines constitutively expressing Dock180/ELMO1 or Dock180/ELMO1 Y724F

**Fig. 4:**
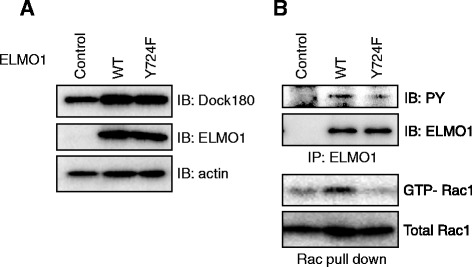
Establishment of NIH3T3-Dock180-ELMO1 cells. **a** NIH3T3 cells stably expressing extrinsic Dock180 were infected with retroviruses encoding wild-type ELMO1 and its Y724F mutant, and the introduced cells were selected with Blasticidin. Expression levels of extrinsic Dock180 and ELMO1 were examined by immunoblotting using Abs to Flag and ELMO1, respectively. **b** ELMO1 phosphorylation and Rac1 activation in NIH3T3-Dock180-ELMO1 cells. Trypsinized NIH3T3-Dock180-ELMO1 cells were replaced onto fibronectin-coated dishes and incubated for 30 min. To examine phosphorylation of ELMO1, immunoprecipitation and the subsequent immunoblotting were performed using Abs to ELMO1 and PY, respectively (upper two panels). For Rac1 activity, the cell lysates were subjected to the pull-down assay, followed by the immunoblotting using anti-Rac1 Ab (bottom two panels)

To investigate the role of ELMO1 phosphorylation in biological responses, we established NIH3T3 cells constitutively expressing both Dock180 and wild-type ELMO1, or both Dock180 and ELMO1 Y724F mutant (Fig. 4a). To confirm the relevance of pY724 of ELMO1 for Rac1 activity in these cells, Rac1 activity upon cell adhesion to the fibronectin-coated dish was examined because fibronectin-induced integrin signalling has been shown to be mediated through the Src, Dock180, and the ELMO1-dependent pathway [22]. The phosphorylation of ELMO1, together with subsequent activation of Rac1, could be observed in NIH3T3 cells expressing wild-type ELMO1, but not the ELMO1 Y724F mutant (Fig. 4b).

### Requirement for phosphorylation of ELMO1 Y724 in cell spreading and adhesion

**Fig. 5:**
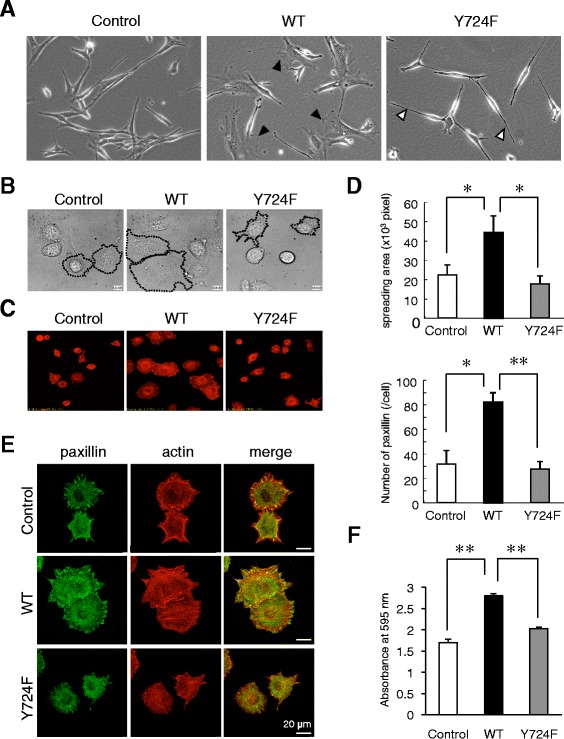
Phosphorylation of ELMO1 Y724 contributes to the enhancement of cell adhesion. NIH3T3-Dock180-Control, −wild-type ELMO1, and - Y724F ELMO1 cells were employed throughout the analyses in Fig. 4. **a** Representative cell morphologies onto the conventional culture dish are shown. Black and white arrowheads indicate the apparent membrane ruffles and the protrusion of the cell membrane, respectively. **b-d** The trypsinized cells were replaced onto glass-based dishes coated with 10 μg/ml fibronectin. **b** Immediately after re-plating, DIC images of cells were obtained every one minute for 1 h using a time-lapse imaging system. The micrographs after 30 min are shown. To define the cell size, the margins of several cells are marked with dotted lines. **c** Cells at 30 min after re-plating were subjected to actin staining. **d** In immunofluorescence described in Fig. 5c and 5e, the sizes of the individual cells (left panel) and the numbers of paxillin (right panel) were determined using MetaMorph software and manually, respectively. The data are shown as mean ± SD of six cells from a single experiment. *, *P* < 0.001; **, *P* < 0.00001. **e** Cells at 30 min after re-plating were subjected to immunofluorescence analysis with Ab to paxillin and with phalloidin for actin staining. In D, representative micrographs are shown and the merged images placed at the right. **f** The cells were replaced onto the 96-well plates coated with fibronectin. After incubation for 90 min, the bound cells were stained, lysed, and measured as described in Materials and Methods. The data are shown as mean ± SD of 3 wells on each condition. *, *P* < 0.005; **, *P* < 0.0001

Under a forced expression of Dock180, the cells with extrinsic wild-type ELMO1 displayed an enlarged cytoplasm with the frequent appearance of membrane ruffles (Fig. 5a, black arrowheads). No significant difference was observed between control cells and the Y724F mutant, except for in the latter only small cell protrusions (Fig. 5a, white arrowheads).

Following cell adhesion, the dynamics of morphological changes were analyzed by time-lapse imaging. Speed and extent of cell spreading on the fibronectin-coated dish was remarkably facilitated by wild-type ELMO1 (Fig. 5b, Additional file 3: Movie S1 vs Additional file 4: Movie S2). Only random and transient protrusions of the cell membrane were observed in the Y724F cells (Fig. 5b, Additional file 5: Movie S3). Actin staining of the cells at 30 min after the plating revealed that the size of spread cell became significantly larger by wild-type ELMO1 (Figs. 5c,d upper panel). Focal adhesions were strongly developed at the end of actin stress fibers in the cells with wild-type ELMO1; indeed, the numbers of focal adhesions, as visualized with an anti-paxillin antibody, were 2.6-fold higher than those in control cells (Figs. 5e,d lower panel). The cells with ELMO1 Y724F displayed insufficient membrane ruffles with faint staining for paxillin (Figs. 5d,e). In agreement with these findings, the ability of cell adhesion was significantly up-regulated by the expression of wild-type ELMO1, on extracellular matrices such as fibronectin (Fig. 5f). Taken together, the pY724 of ELMO1 plays a critical role in organizations of the actin cytoskeleton and focal adhesion, and the subsequent cell adhesion.

### Y724 phosphorylation of ELMO1 facilitates cell migration

**Fig. 6:**
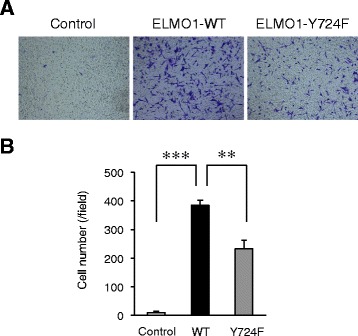
ELMO1 pY724 facilitates cell migration. **a** Trans-well migration assay. NIH3T3-Dock180-Control, −wild-type ELMO1, and -Y724F ELMO1 cells were suspended in DMEM without FBS were seeded into the upper chamber. DMEM containing 10 % FBS was added to the lower chamber. After 11 h, migrating cells were stained with 0.04 % crystal violet. Representative micrographs after incubation for 11 h are shown. **b** The motility of the individual cells was evaluated by measuring the migrating cells. The data are shown as mean ± SD. **, *P* < 0.005; ***, *P* < 0.0005

To clarify whether pY724 of ELMO1 is involved in cell migration, we performed a trans-well migration assay using Boyden chamber in NIH3T3 cells with wild-type ELMO1 or its Y724F mutant, upon extrinsic Dock180 expression. We succeeded to detect the strikingly enhancement of the motility upon WT-ELMO1 expression, and significant decrease by the Y724F mutation (Figs. 6a,b). It should be noted that in wound healing assay, we could not observe the inhibitory effect by the Y724F mutant, with or without extrinsic Dock180 (Additional file 6: Figure S2, Additional file 7: Text S1, and Additional file 8: Figure S3). We also performed a phagokinetic track assay to visualize the random motility at a single cell level (Additional file 7: Text S1). Forced expression of wild-type ELMO1 did not enhance cell motility (Additional file 9: Figure S4), indicating the sufficient amount of intrinsic ELMO1 for collaborating with Dock180 under this condition. Meanwhile, the cells with ELMO1 Y724F mutant significantly reduced cell migration regardless of fibronectin coating (Additional file 9: Figure S4).

## Discussion

ELMO1 has been shown to functionally cooperate with CrkII and Dock180 to regulate Rac1 activity during cell migration and phagocytosis [3]. In the present study, we have demonstrated that Src-mediated phosphorylation of Y724 of ELMO1 regulates Rac1 activation, and promotes cell adhesion and motility. Whereas forced expression of Hck also leads to tyrosine phosphorylation of ELMO1 on Y216, Y395, Y511, and Y720, we here identified 724 residue of ELMO1 as a preferential phosphorylation sites by Src.

The significance of the complex formation of ELMO1 and Dock180 in Rac1 activation has been reported. Previous study disclosed the C-terminal polyproline region of ELMO1 (707 to 714 amino acids) essentially interacts with the SH3 domain of Dock180 [6]. This region may serve also as a potential binding site for Src through its SH3 domain. Indeed, the C-terminal region of ELMO1 (663–724) is sufficient to be phosphorylated by Src (Fig. 2d). The phosphorylation of Y724 evoked by Src might be dispensable for the binding of ELMO1 and Dock180, because ELMO1 appeared to be able to form the complex with Dock180 in the absence of active Src (Fig. 3), in which ELMO1 received no phosphorylation (Fig. 2c). In fact, an accumulation of Dock180 was observed upon co-expression of ELMO1 and Dock180 in a Src-independent fashion, irrespective of the phosphorylation status of Y724 (Fig. 3, lanes 1–3). These results indicate the prevention of Dock180 ubiquitination owing to the interaction with ELMO1, as we have reported [14].

We demonstrated that phosphorylation of Y720 and Y724 seem to be reciprocally dependent on each other (Fig. 2d). Because the pY720 provides the SH2 binding sequence (pTyr-Asp-Phe-Val), SFK including Src binds to pY720 and progressively phosphorylates Y724 of ELMO1 (Additional file 10: Figure S5). In agreement with this consideration, Y724F mutation may influence on the phosphorylation status of Y720, and thereby we should pay attention to this point in any mutational analysis. Meanwhile, the Hck was previously reported to phosphorylate Y511 of ELMO1, but as the expression of Hck is restricted in hematopoietic lineages, a tissue-specific phosphorylation mode of ELMO1 may be present. Ultimately, different SFKs could regulate distinct cellular functions in diverse cell types through ELMO1 phosphorylation. The detailed molecular mechanism(s) underlying how the phosphorylation at the C-terminus of ELMO1 augments Rac1 activity remains to be investigated in the future. We are hypothesizing that phosphorylated ELMO1 recruits additional elements such as Crk and ERM, and modulates downstream signaling of Rac1 [23].

Disregulation of Src has been observed in a variety of human malignancies [21]. It has been proposed that Src functions in cooperation with diverse signalling derived from tumor microenvironment [24]. It is well established that the extracellular matrix (ECM) contributes to the invasion of tumor cells, in which cell-to-ECM adhesion through integrins is the first crucial step. In this context, Src plays a prominent role in the regulation of cell adhesion, migration, and invasion *via* interaction with integrins, Rho GTPase, and focal adhesion components including p130^Cas^, paxillin, and FAK [22]. Specifically, signals derived from αvβ5 and β1 integrins have been shown to induce Src-dependent phosphorylation of p130^Cas^ , which leads to the recruitment of CrkII/Dock180 complex and subsequent Rac activation [25–27]. Our observation may provide an additional molecular mechanism underlying the Src mediated local activation of Rac1. Corresponding to Src activity arising from ECM adhesion, Dock180 is recruited, and Dock180 is further activated through Src-dependent phosphorylation on Y724 of ELMO1, which may contribute to achieve highly polarized Rac activity (Additional file 9: Figure S4). In fact, it was reported that in migrating or PDGF-stimulated cells, the gradient of Rac activity is more polarized compared to that of Src, despite the Src-Rac hierarchy in their regulation [28]. Meanwhile, a ternary complex comprised of RhoG-ELMO1-Dock180 mediates integrin-induced cell spreading of HeLa cells [29]. As RhoG recruits ELMO1/Dock180 complex at plasma membrane for efficient Rac1-dependent cell spreading on fibronectin [9, 30], the facilitation of the complex formation owing to Src-dependent pY724 of ELMO1 might be beneficial to rapid response to integrin stimulation (Additional file 10: Figure S5).

For tumor microenvironment, the stromal cells were approved as an ideal source of exogenous stimuli for tumor cells by producing numerous cytokines, steroid hormones, and other extrinsic factors that affect cancer cell motility. As these extrinsic factors could induce the activation of Src [22, 31], they might also enhance migration of tumor cells through possible Y724 phosphorylation of ELMO1.

On the other hand, the ELMO1/Dock180 complex is an important regulator of Rac1 activation in endothelial cells *in vitro* and during the formation of the vasculature in zebrafish *in vivo* [32]. These findings might represent the consequence of this complex also in cell adhesion and migration of endothelial cells during tumor angiogenesis, because an activation of Src has shown in endothelial cells of tumor tissues.

Recent studies highlight Src as a potent target for therapeutic intervention; especially as the targeting can overcome multiple mechanisms of trastuzumab resistance in breast cancer therapeutics, because hyperactivation of Src is universally detected in the resistant cells [31, 33]. As an activation of Src leads to an elevation of the complex consisting of p130^Cas^/Crk/Dock180 in breast cancer cells [34], ELMO1/Dock180 binding must be important for producing Src-dependent activation of Rac1. Because aberrant activation of cell motility pathways may underlie the variety in cancer cell invasion, the elucidation of mechanisms underlying ELMO1 pY724-mediated promotion in Rac activity might establish these molecules as potential targets for effective cancer therapies.

## Conclusions

In summary, we provide evidences that Src-mediated tyrosine phosphorylation of ELMO1 at Y720 and Y724 residues, which is crucial for activation of Rac1, and subsequent cell adhesion, spreading, and migration. As the overexpression and/or hyperactivation of Src have been shown in a wide variety of human cancers, Src-mediated phosphorylation of Y724 in ELMO1 may regulate cancer cell adhesion to the extracellular matrix, invasion into surrounding tissues, and subsequent distant metastasis.

## Materials and methods

### Cell culture

HEK293T and NIH3T3 cells were cultured in Dulbecco’s Modified Eagle’s Medium (DMEM, Nissui, Japan) supplemented with 10 % fetal bovine serum (FBS, Biosource, Camarillo, CA, USA) (complete DMEM). NIH3T3 cells expressing Dock180 and ELMO1 were maintained in complete DMEM with 500 μg/ml G418 (Sigma, St. Louis, MO, USA) and 10 μg/ml Blasticidin S-HCl (Invitrogen, Carlsbad, CA, USA), respectively, and cells expressing both Dock180 and ELMO1 were maintained in complete DMEM containing both of the antibiotics. All cells were maintained under conditions of 5 % CO_2_ at 37 °C.

### Antibodies and immunoblotting

Antibodies were purchased from suppliers as follows: antibodies to Flag (M2PO) were from Sigma; those to phosphotyrosine (PY20H) and Rac1 (102) were from BD Transduction Laboratories (Lexington, KY, USA); those to GST was from GE Healthcare (Buckinghamshire, UK); those to actin (C4) was from Chemicon International (Temecula, CA, USA); those to Src (GD11) was from Upstate Biotechnology (Lake Placid, NY, USA); those to non-phospho-Src Y416 was from Cell Signaling Technology (Beverly, MA, USA); those to Myc (9E10) was from Invitrogen; and those to Elmo1 (clone ab2239) was from Abcam (Cambridge, UK). Immunoblotting was performed as described previously [14].

### Plasmids

pCXN2-Flag-Dock180 and pEBB-Flag-Elmo1 were kind gifts from Dr. M. Matsuda (Kyoto Univ., Kyoto, Japan) and Dr. KS. Ravichandran (Univ. of Virginia, Charlottesville, VA, USA), respectively. pCXbsr and pCLEco were from Dr. T. Akagi (Kan Institute, Japan), and pMik-Src and pMik-Src-Y527F, and mammalian expression plasmids for Fyn, Yes, Lck, Lyn, Hck were from Dr. H. Hanafusa (Rockefeller Univ., NY, USA). pCMV-myc-Elmo1 was constructed as described previously [14]. The mutant forms of pCMV-myc-Elmo1 such as Y60F, Y216F, Y352F, Y356F, Y395F, Y511F, Y646F, Y662F, Y720F, Y724F and Y720/724 F (double mutant) were constructed by reverse-PCR-based method using pCMV-myc-Elmo1 as a template. The cDNA of Elmo1 and Src Y527F were amplified by PCR using pEBB-Flag-ELMO1 and pMik-Src Y527F as a template, respectively, and subcloned into pCXN2-Flag and pGEX vectors at XhoI/NotI sites; the resulting plasmids were named pCXN2-Flag-Elmo1, pGEX-Elmo1, and pCXN2-Flag-Src Y527F. The DNA fragments encoding 663–727 amino acid of Elmo1 were amplified by PCR from pCMV-myc-Elmo1 and its mutant forms of Y720F, Y724F, and Y720/724 F as a template, and inserted into pEBG vector at XhoI/NotI sites, and the resultant plasmids were named pEBG-ElmoΔ662, ElmoΔ662-Y720F, −Y724F, and -Y720/724 F. The cDNA of wild-type Elmo1 and its Y724F mutant amplified by PCR were subcloned into pCXbsr vector at BamHI/NotI sites, and the resultant constructs were named pCXbsr-Elmo1 and -Elmo Y724F. All PCR fragments were verified by sequencing.

### Establishment of NIH3T3 cell lines expressing Dock180, ELMO1, and both Dock180 and ELMO1

NIH3T3 cells were transfected with pCXN2-Flag-Dock180 using Lipofectamine 2000 (Invitrogen), and selected by 500 μg/ml G418 (Sigma). The obtained clones were screened by immunoblot analysis using anti-Flag antibody, and the successfully resulting cell line constitutively expressing Dock180 was named NIH3T3-Dock180. To establish NIH3T3 cells expressing wild-type ELMO1 and its Y724F mutant, BOSC23 cells were transfected with pCXbsr-empty, −Elmo1, or -Elmo1 Y724F in combination with pCLEco vector, and the supernatant containing the corresponding retroviruses were inoculated to NIH3T3 and NIH3T3-Dock180 cells. Cells were then incubated in the presence of 10 μg/ml Blasticidin S-HCl, and the established cell lines were named NIH3T3-Control, NIH3T3-ELMO1 wild type, NIH3T3-ELMO1 Y724F, and NIH3T3-Dock180/Control, NIH3T3-Dock180/ELMO1 wild type, NIH3T3-Dock180/ELMO1 Y724F. The expression levels of exogenous proteins were verified by immunoblotting.

### Detection for tyrosine phosphorylation of ELMO1 *in vitro* and *in vivo*

For *in vitro* phosphorylation assay, recombinant proteins of both c-Src (Invitrogen) and ELMO1 which was purified from the E.coli BL21 strain transformed with pGEX-ELMO1, were mixed in kinase buffer (40 mM HEPES-NaOH (pH 7.4), 10 mM MgCl_2_, 1 mM DTT, with or without 10 μM ATP), and incubated at 25 °C for 5 min. ELMO1 phosphorylated by c-Src was detected by immunoblotting with anti-phosphotyrosine antibody. For *in vivo* phosphorylation assay, HEK293T cells were transfected with the plasmids expressing ELMO1 and one of the Src family kinases. After 48 h, the cells were lysed and subjected into immunoprecipitation with antibodies to Flag, Myc, or ELMO1, and the precipitants were analyzed by immunoblotting using anti-phosphotyrosine antibody.

### Pull-down assay for Rac1 activity

HEK293T cells were lysed with buffer containing 1 % NP-40, 25 mM HEPES (pH 7.4), 150 mM NaCl, 10 % (v/v) glycerol, 1 mM EDTA, 10 mM MgCl_2_, 1 mM PMSF, and a complete protease inhibitor cocktail, and the lysates were centrifuged at 12,000 rpm at 4 °C for 1 min. The supernatants were incubated with 10 μg of purified GST-PAK2-RBD, and then with glutathione-Sepharose 4B beads (GE Healthcare, Little Chalfont, UK). Following the washing of beads three times, the precipitants were analyzed by immunoblotting with anti-Rac1 antibody.

### Cell spreading assay by time lapse imaging and immunofluorescence

NIH3T3-Dock180-Control, NIH3T3-ELMO1 wild type, or NIH3T3-ELMO1 Y724F cells were trypsinized and suspended in complete DMEM for 30 min. The cells were subsequently seeded onto fibronectin-coated glass-based dishes. Cell spreading was evaluated by time-lapse-based imaging technique and immunofluorescence.

For time-lapse imaging analysis, immediately after cell inoculation, differential interference contrast (DIC) images of the cells were obtained every one minute for 1 h using a time-lapse system of an Olympus IX-70 inverted microscope (Tokyo, Japan), a Photometrics cooled charge-coupled device camera (Tucson, AZ, USA), and a Ludl mechanical shutter, which were controlled by MetaMorph software (Universal Imaging, Downingtown, PA, USA).

For immunofluorescence analysis, after 30 min of cell plating, the fixation and staining for actin and paxillin were performed as described previously [35]. Fluorescence images were obtained using a confocal laser-scanning microscope (FV-300; Olympus, Tokyo, Japan). The spreading area of the cells was analyzed by MetaMorph software and the numbers of positive dots of paxillin staining were manually counted.

### Cell adhesion assay

The NIH3T3 cells utilized in cell spreading assay were also employed for cell adhesion analysis. 2 × 10^4^ cells suspended in DMEM containing 0.02 % bovine serum albumin (BSA) were plated into each well of a 96-well microplate coated with 10 μg/ml fibronectin (Biomedical Technologies Inc., Stoughton, MA, USA). After incubation at 37 °C for 90 min under a humidified atmosphere containing 5 % CO_2_, the bound cells were stained with 0.04 % crystal-violet in DMSO, and quantified by measuring the absorbance at 595 nm wave with a spectrophotometer (Thermo Electron Corporation, Vantaa, Finland).

### Trans-well migration assay

For the trans-well migration assay using Boyden chamber, 2.5 × 10^**4**^ cells of NIH3T3-Dock180-Control, NIH3T3-Dock180-ELMO1 wild type, or NIH3T3-Dock180-ELMO1 Y724F were suspended in DMEM without FBS were seeded into the upper chamber. DMEM containing 10 % FBS was added to the lower chamber as chemoattractant. After incubation for 11 h, the non-migrating cells on the upper surface of the filters were removed by a wiping with a cotton swab. Migrating cells were fixed with 3.7 % paraformaldehyde in PBS, stainined with 0.04 % crystal violet, and counted. Data represent means and standard deviations from a single experiment, and were subject to two-way analysis of variance, followed by the comparison by Student’s t-test. *P* values obtained from the test are described in the figure legends.

## Additional files

Additional file 1: Table S1. Predictions for tyrosine phosphorylation of ELMO1 were shown. Additional file 2: Figure S1. Rac1 activity in cells with defective tyrosine of ELMO1. Lysates from HEK293T cells transfected with pCMV-Myc-ELMO1 or -its defective mutants in combination with pCXN2-Flag-Dock180 were subjected into pull-down assay for Rac1 activity. Additional file 3: Movie S1. NIH3T3-Dock180-Control cells were employed for cell spreading assay. The trypsinized cells were replaced onto glass-based dishes coated with 10 μg/ml fibronectin. Immediately after re-plating, DIC images of cells were obtained every one minutes for 1 h using a time-lapse imaging system. Additional file 4: Movie S2. NIH3T3-Dock180-wild-type ELMO1 cells were employed for cell spreading assay described in Additional file 3: Movie S1, and movie of the DIC images were shown. Additional file 5: Movie S3. NIH3T3-Dock180-ELMO1 Y724F cells were employed for cell spreading assay described in Additional file 3: Movie S1, and movie of the DIC images were shown. Additional file 6: Figure S2. NIH3T3-ELMO1 cells were established by infection of retroviruses bearing Control, ELMO1, and its Y724F mutant. Expression levels of ELMO1 were examined by immunoblotting. Additional file 7: Text S1. Materials and methods for Wound-healing assay and Phagokinetic track assay were described. Additional file 8: Figure S3. Wound-healing assay using NIH3T3-ELMO1 cells (A) and NIH3T3-Dock180-ELMO1 cells (B) are shown. Scale bars: 500 μm. Additional file 9: Figure S4. ELMO1 pY724 facilitates cell migration. (A) Analysis of cell motility by phagokinetic track assay. NIH3T3-Control, −wild-type ELMO1, and -Y724F ELMO1 cells were placed onto gold particles-mounted cover glasses coated with or without 10 μg/ml fibronectin. Representative micrographs after incubation for 20 h are shown. (B) The motility of the individual cells was evaluated by measuring the area cleared of gold particles using MetaMorph software. The data are shown as mean ± SD of 30 cells on each condition. *, *P* < 0.001; **, *P* < 0.0001; ***, *P* < 0.0000001. Additional file 10: Figure S5. Schematic representation of summary of this study combined with previous findings. (A) Without Src activation. SH3 domain of Dock180 intramolecularly binds to the Docker domain, thereby preventing Rac from accessing to the Docker domain. (B) With Src activation. In previous findings, following ECM adhesion such as fibronectin, Src phosphorylates focal adhesion molecules such as p130^Cas^ and paxillin (black arrows), which leads to the recruitment of CRK/Dock180 complex and subsequent Rac activation. Here, we identified that Src directly phosphorylates ELMO1 at Y720 and Y724 residues (red arrows), which enhances the interaction between ELMO1 and Dock180 *via* Pro-rich region and the SH3 domain, respectively. As RhoG has shown to recruit ELMO1/Dock180 complex at plasma membrane, the facilitation of ELMO1/Dock180 complex formation owing to the Src-dependent pY720/pY724 of ELMO1 might be beneficial to rapid response to integrin stimulation and highly polarized Rac activation, which contribute to subsequent cell adhesion, spreading, and migration.

## Abbreviations

Dock180: Downstream of Crk with 180 kDa
ELMO1: Regulator of engulfment and motility 1
SFKs: Src family of protein tyrosine kinases
Crk: CT10 regulator of kinase
GEF: Guanine nucleotide exchange factor
PH: Pleckstrin homology
ARM: Armadillo
Hck: Hematopoietic cell kinase
ECM: Extracellular matrix
DIC: Differential interference contrast
