# Supplementary material for: Tyr724 phosphorylation of ELMO1 by Src is involved in cell spreading and migration via Rac1 activation
Source: Cell Commun Signal. 2015 Jul 25;13:35. doi: 10.1186/s12964-015-0113-y (PMC4513707; doi:10.1186/s12964-015-0113-y)

# Additional file 1: Table S1. Predictions for tyrosine phosphorylation of ELMO1. Amino acid sequence of ELMO1 was applied to NetPhos service (CBS Prediction Servers, Technical Univ. of Denmark) and the predictive results are shown in the Table. Position: position of tyrosine residue in ELMO1, Context: sequence around each tyrosine residue, Score: predicted possibility of tyrosine phosphorylation, Pred: predicted phosphorylation sites were indicated by *Y*.


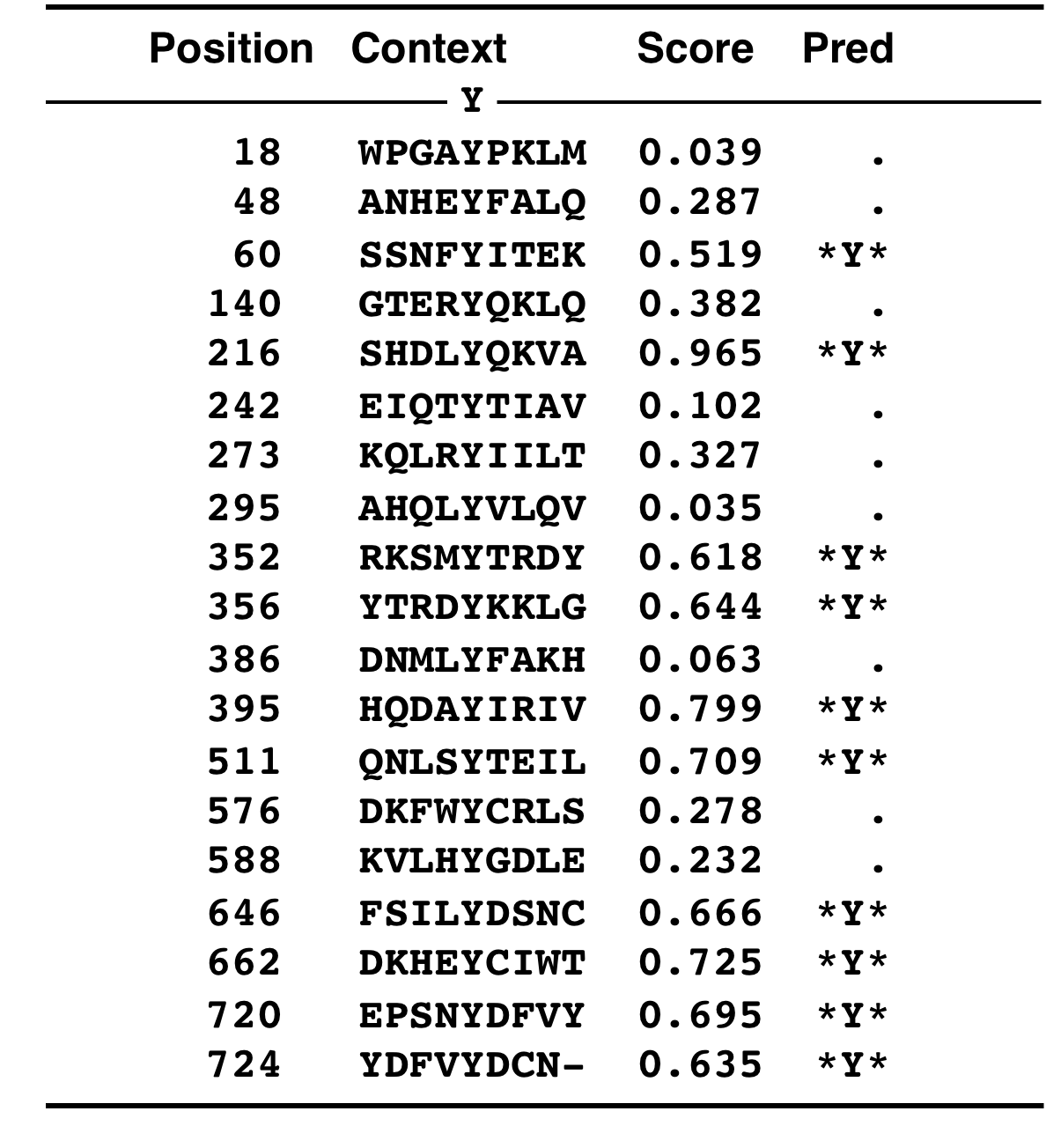

Supplement: Additional file 1: Table S1. — Predictions for tyrosine phosphorylation of ELMO1 were shown. [file 12964_2015_113_MOESM1_ESM.docx]
