# Supplementary figures and images for: Tyr724 phosphorylation of ELMO1 by Src is involved in cell spreading and migration via Rac1 activation
Source: Cell Commun Signal. 2015 Jul 25;13:35. doi: 10.1186/s12964-015-0113-y (PMC4513707; doi:10.1186/s12964-015-0113-y)

Additional file 2: Figure S1

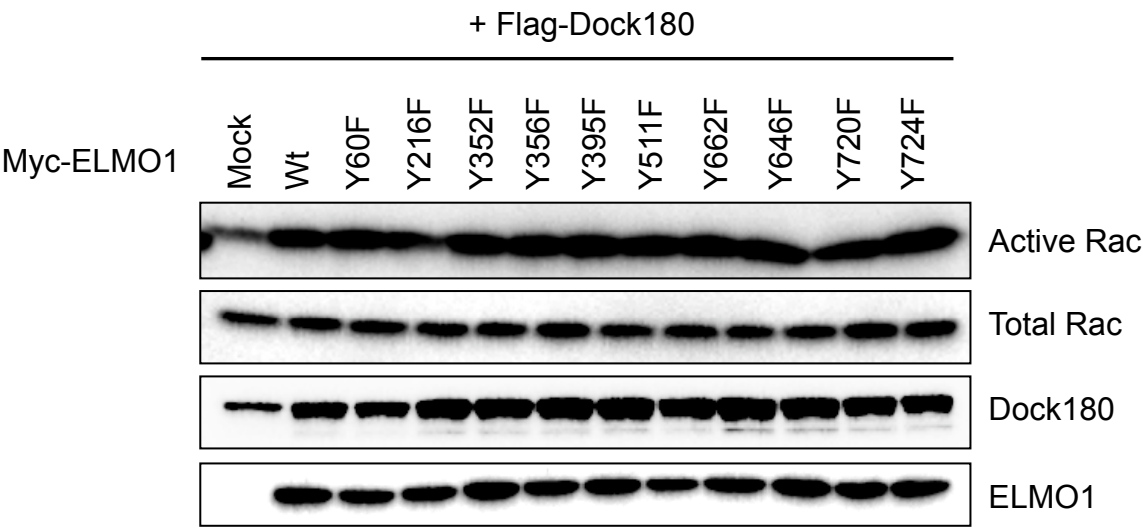

Makino et al.

Supplement: Additional file 2: Figure S1. — Rac1 activity in cells with defective tyrosine of ELMO1. Lysates from HEK293T cells transfected with pCMV-Myc-ELMO1 or -its defective mutants in combination with pCXN2-Flag-Dock180 were subjected into pull-down assay for Rac1 activity. [file 12964_2015_113_MOESM2_ESM.pdf]

## Additional file 6: Figure S2

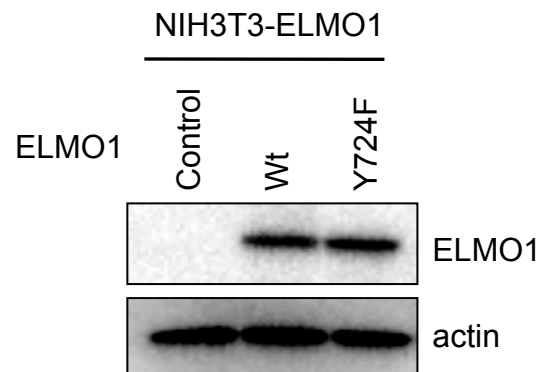

**Makino et al.**

Supplement: Additional file 6: Figure S2. — NIH3T3-ELMO1 cells were established by infection of retroviruses bearing Control, ELMO1, and its Y724F mutant. Expression levels of ELMO1 were examined by immunoblotting. [file 12964_2015_113_MOESM6_ESM.pdf]

**Additional file 8: Figure S3**

**A** NIH3T3-ELMO1

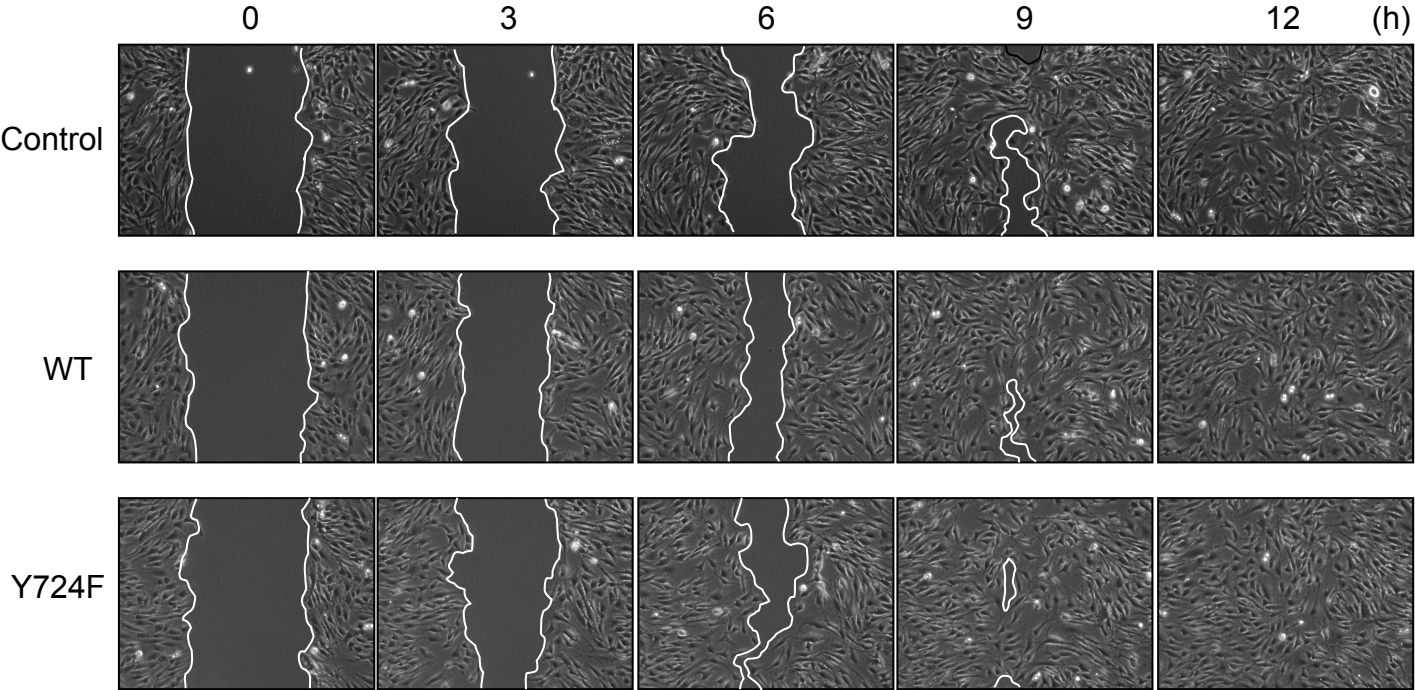

**B** NIH3T3-Dock180-ELMO1

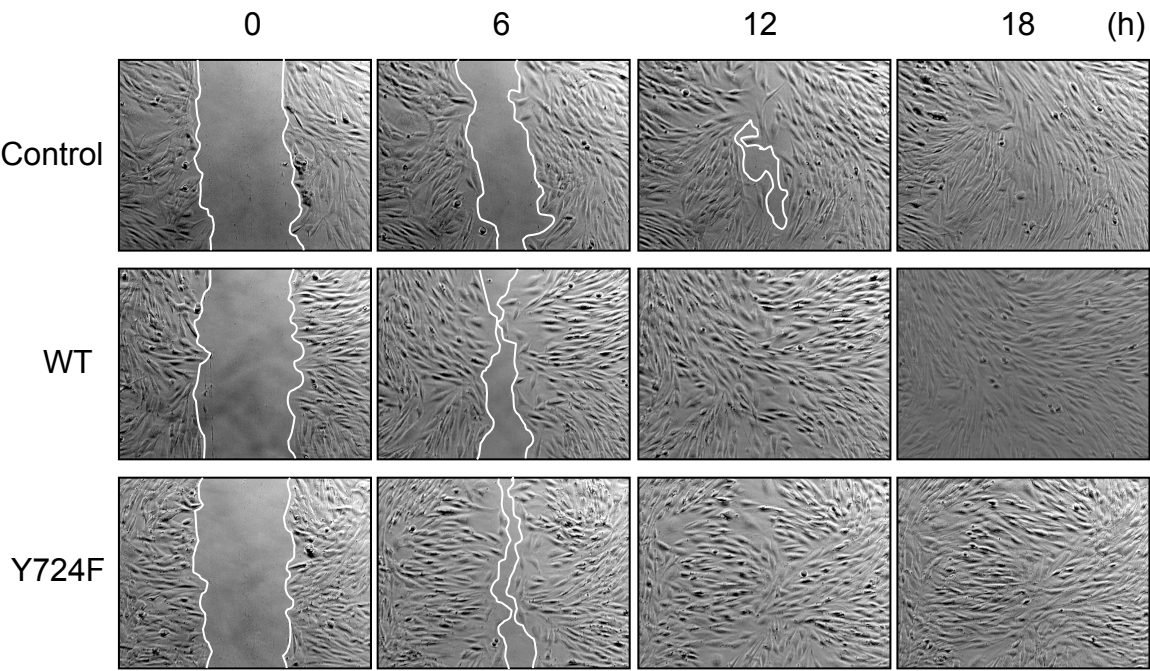

Supplement: Additional file 8: Figure S3. — Wound-healing assay using NIH3T3-ELMO1 cells (A) and NIH3T3-Dock180-ELMO1 cells (B) are shown. Scale bars: 500 μm. [file 12964_2015_113_MOESM8_ESM.pdf]

Additional file 9: Figure S4

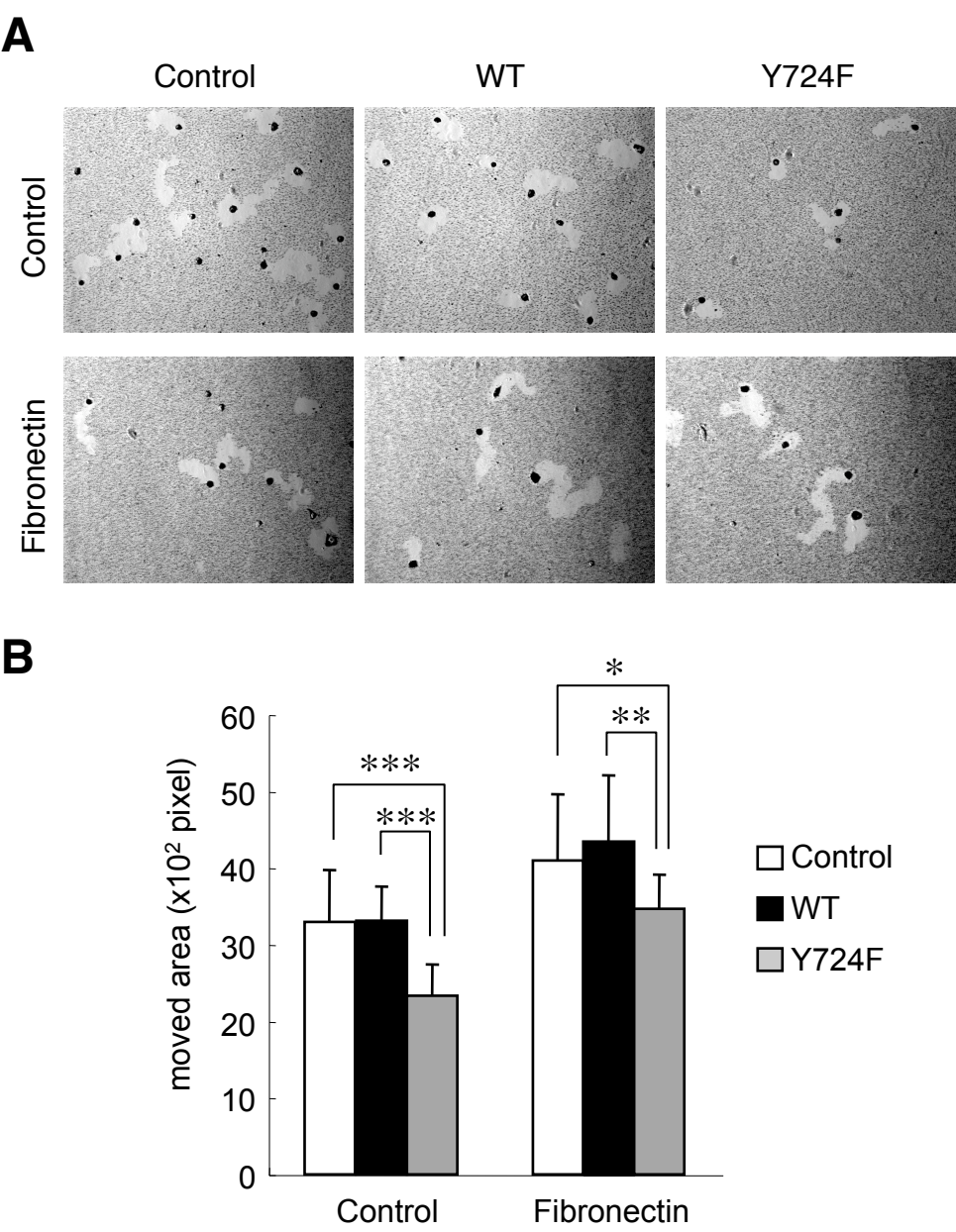

Supplement: Additional file 9: Figure S4. — ELMO1 pY724 facilitates cell migration. (A) Analysis of cell motility by phagokinetic track assay. NIH3T3-Control, −wild-type ELMO1, and -Y724F ELMO1 cells were placed onto gold particles-mounted cover glasses coated with or without 10 μg/ml fibronectin. Representative micrographs after incubation for 20 h are shown. (B) The motility of the individual cells was evaluated by measuring the area cleared of gold particles using MetaMorph software. The data are shown as mean ± SD of 30 cells on each condition. *, P < 0.001; **, P < 0.0001; ***, P < 0.0000001. [file 12964_2015_113_MOESM9_ESM.pdf]

**Additional file 10: Figure S5**

**A** Without Src activation

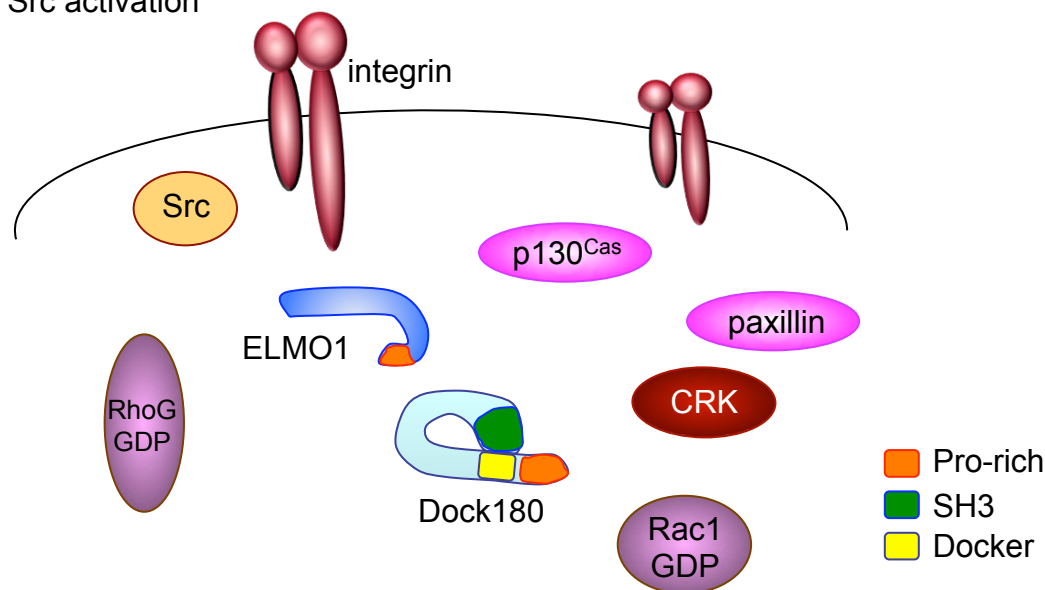

**B** With Src activation

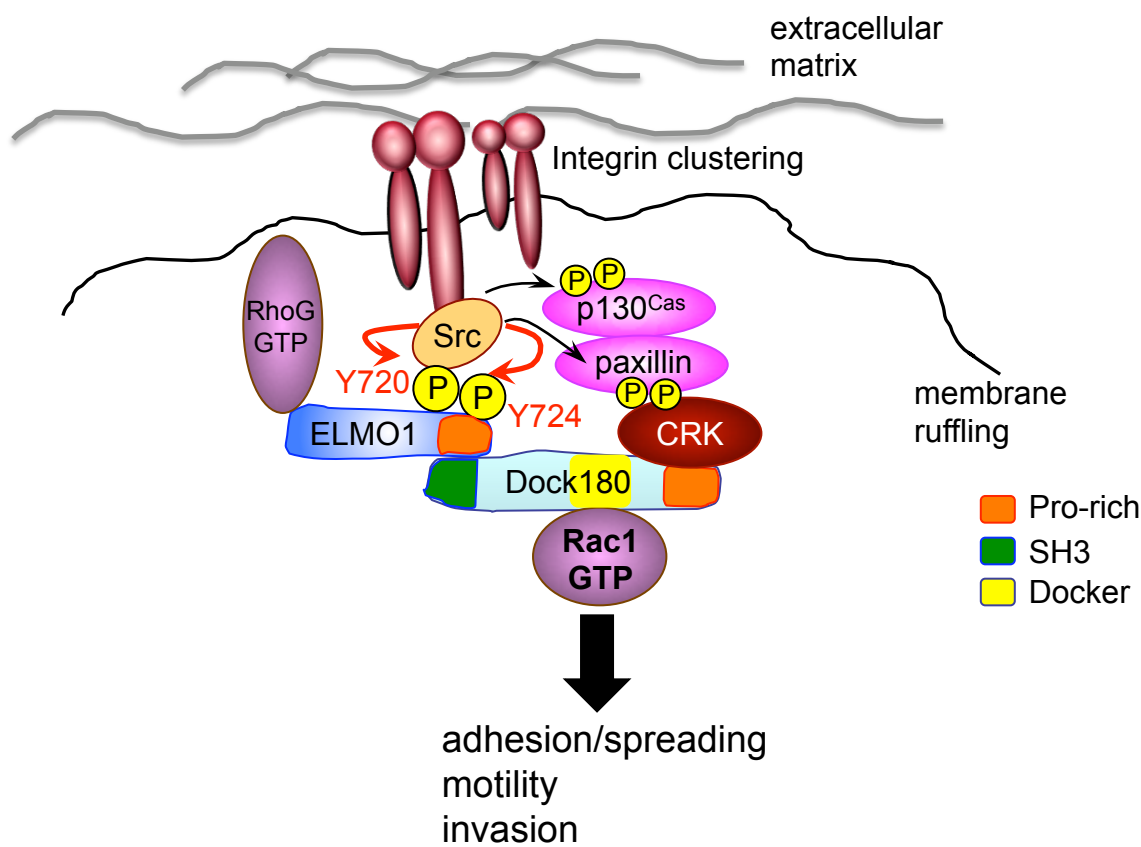

**Makino et al.**

Supplement: Additional file 10: Figure S5. — Schematic representation of summary of this study combined with previous findings. (A) Without Src activation. SH3 domain of Dock180 intramolecularly binds to the Docker domain, thereby preventing Rac from accessing to the Docker domain. (B) With Src activation. In previous findings, following ECM adhesion such as fibronectin, Src phosphorylates focal adhesion molecules such as p130Cas and paxillin (black arrows), which leads to the recruitment of CRK/Dock180 complex and subsequent Rac activation. Here, we identified that Src directly phosphorylates ELMO1 at Y720 and Y724 residues (red arrows), which enhances the interaction between ELMO1 and Dock180 via Pro-rich region and the SH3 domain, respectively. As RhoG has shown to recruit ELMO1/Dock180 complex at plasma membrane, the facilitation of ELMO1/Dock180 complex formation owing to the Src-dependent pY720/pY724 of ELMO1 might be beneficial to rapid response to integrin stimulation and highly polarized Rac activation, which contribute to subsequent cell adhesion, spreading, and migration. [file 12964_2015_113_MOESM10_ESM.pdf]
