# Supplementary material for: Tyr724 phosphorylation of ELMO1 by Src is involved in cell spreading and migration via Rac1 activation
Source: Cell Commun Signal. 2015 Jul 25;13:35. doi: 10.1186/s12964-015-0113-y (PMC4513707; doi:10.1186/s12964-015-0113-y)
Supplement: Additional file 7: Text S1. — Materials and methods for Wound-healing assay and Phagokinetic track assay were described. [file 12964_2015_113_MOESM7_ESM.docx]

# Additional file 7: Text S1. Supporting information

# Supplementary Materials and Methods

Wound-healing assay. NIH3T3-ELMO1 cells and NIH3T3-Dock180-ELMO1 cells grown to confluence on a fibronectin-coated dish were scratched off, and further incubated for 12 h and 18 h, respectively. Images were taken every 3 h (NIH3T3-ELMO1 cells) or 6 h (NIH3T3-Dock180-ELMO1 cells) using a time-lapse system consisting of an Olympus IX-71 inverted microscope (Tokyo, Japan), a Photometrics cooled charge-coupled device camera (Tucson, AZ), and a Ludl mechanical shutter, which were controlled by MetaMorph software (Universal Imaging, Downingtown, PA).

**Phagokinetic track assay.** Random cell motility was assessed by the phagokinetic track assay. Briefly, carpets of gold particles were prepared on cover glasses (18 × 18 mm; Matsunami, Japan) coated with 1% BSA, with or without overcoating of 10 µg/ml fibronectin (Biomedical Technologies, Inc.). Five thousand cells of NIH3T3-Control, NIH3T3-ELMO1 wild type, or NIH3T3-ELMO1 Y724F were plated on the cover glasses. After incubation for 20 h, the cover glasses were fixed in 10% formalin. The phagokinetic tracks were observed under a microscope at a magnification of ×20 objective lens, and the area cleared of gold particles by 30 randomly selected cells in each condition was measured using MetaMorph software. Data represent means and standard deviations from a single experiment, and were subject to two-way analysis of variance, followed by the comparison by Student’s t-test. P values obtained from the test are described in the figure legends.
